# *BRCA1* mRNA expression modifies the effect of T cell activation score on patient survival in breast cancer

**DOI:** 10.1186/s12885-019-5595-3

**Published:** 2019-04-25

**Authors:** Lingeng Lu, Huatian Huang, Jing Zhou, Wenxue Ma, Sean Mackay, Zuoheng Wang

**Affiliations:** 1 Department of Chronic Disease Epidemiology, Yale School of Public Health, School of Medicine, Center for Biomedical Data Science, Yale Cancer Center, Yale University, 60 College Street, New Haven, CT 06520-8034 USA; 2 Guizhou Qianxinan People’s Hospital, Xingyi, 652400 Guizhou China; 3 Isoplexis Corporation, 35 NE Industrial Road, Branford, CT 06405 USA; 4 Moores Cancer Center, University of California San Diego, La Jolla, CA 92093 USA; 5 Department of Biostatistics, Yale School of Public Health, School of Medicine, Center for Biomedical Data Science, Yale Cancer Center, Yale University, 60 College Street, New Haven, CT 06520-8034 USA

**Keywords:** Breast cancer, *BRCA1*, *CCND1*, Prognosis, T cell activation score

## Abstract

**Background:**

Effector CD8^+^ T cell activation and its cytotoxic function to eradicate tumor cells depend on the T cell recognition of tumor neoantigens, and are positively associated with improved survival in breast cancer. Tumor suppressor *BRCA1* and cell cycle regulator *CCND1* play a critical role in maintaining genome integrity and tumorigenesis, respectively. However, it is still unclear how *BRCA1* and *CCND1* expression levels affect the effect of T cell activation on breast cancer patient survival.

**Methods:**

The interactions between T cell activation status and either *BRCA1* or *CCND1* expression were evaluated using Kaplan-Meier survival curves and multivariate Cox regression models in a public dataset with 1088 breast cancer patients.

**Results:**

Among the patients with low *BRCA1* or *CCND1* expression, the Activation group showed better overall survival than the Exhaustion group. Adjusted hazards ratios were 0.43 (95% CI: 0.20–0.93) in patients with a low *BRCA1* level, and 0.39 (95% CI: 0.19–0.81) in patients with a low *CCND1* level, respectively. There was a significant trend in both subgroups (p-trend = 0.011 in the low *BRCA1* group, and p-trend = 0.009 in the low *CCND1* group). In contrast, there is no significant association in patients with either high *BRCA1* or high *CCND1* levels. There is a significant interaction between T cell activation status and *BRCA1* level (*p* = 0.009), but not between T cell activation status and *CCND1* level (*p* = 0.135).

**Conclusions:**

*BRCA1* expression modified the effect of T cell activation status on patient survival in breast cancer, suggesting that the existence of neoantigens and the enhancement of neoantigen presentation in combination with immune checkpoint blockade may have synergistic effects on patient outcome.

## Background

Three key components, tumor infiltrating lymphocytes (TILs), neoantigens, and effector CD8^+^ T cell activation, are involved in the development and progression of human cancers including breast cancer. TILs are a mixture of different types of mononuclear immune cells, mainly dominated by T cells [1, 2]. Infiltrated T cells are primed and activated by tumor neoantigens to be effector T cells, which execute their cytotoxic activities to eliminate tumor cells. Neoantigens are neo-epitopes solely raised in tumor cells, and are generated by tumor-specific DNA alterations that lead to the change of protein sequences. These alterations are one of the key features triggering antitumor immunity. Based on whole-exome sequencing data from 19 types of human cancer, Turajlic et al. [3] found insertion/deletion and non-synonymous mutations in pan-cancer patients. They also demonstrated that the presence of mutation-specific neoantigens was positively associated with T cell activation and checkpoint inhibitor response [3]. Neoantigens bind to MHC class I molecules and are recognized by CD8^+^ T cells. Thus, the efficacy of checkpoint immunotherapy, adoptively transferred T cells and engineered chimeric antigen receptor T (CAR-T) cells depends on the presence of neoantigens on tumor cells [4–8]. However, during the co-evolution of tumor and stromal cells, immunosuppressive microenvironments are fostered, and effector CD8^+^ T cells are exhausted, resulting in the escape of tumor cells from the immune surveillance.

A battery of inhibitory molecules are associated with effector CD8^+^ T cell exhaustion [9]. Cytotoxic T lymphocyte antigen 4 (CTLA4) and programmed cell death-1 receptor (PD-1) are two extensively investigated inhibitors that dampen the function of effector T cells by engaging as receptors of their ligands [10]. Immune checkpoint-based immunotherapies benefit patient’s outcomes by reinvigorating effector CD8^+^ T cells to destroy tumor cells. Anti-CTLA4 (ipilimumab, tremelimumab) and anti-PD-1 (nivolumab, pembrolizumab) immunotherapies have been approved by the United States Food and Drug Administration (FDA) for different types of human cancer. An objective response rate of 18.5% was observed in a phase I trial of anti-PD-1 antibodies in PD-L1-positive triple-negative breast cancer patients [11], which indicates that a substantial proportion of patients in trials did not respond well to the checkpoint immunotherapies. Two possibilities, if not all but at least, may explain the failure to respond to the checkpoint immunotherapies. One is that other inhibitory molecules beyond CTLA4 and PD-1, for example, TIM3, LAG3 and TIGIT, are directly or indirectly, but synergistically involved in the exhaustion of effector T cells [12, 13]. Another possibility is the insufficient amount of neoantigens on tumor cells. Recently, FDA approved the first monoclonal antibody of pembrolizumab (Keytruda) for solid human cancer with DAN repair deficiency. Patients with DNA repair deficiency show significant improvement in both the objective response rate and the survival rate when compared to those with DNA repair proficiency [14–16]. Several case reports show that patients who have better response to immune checkpoint blockade carry higher levels of MHC class I molecule in microsatellite unstable tumors [17–19]. These findings warrant further studies to investigate how neoantigen loads in tumors affect patients’ response to immunotherapies.

*BRCA1* is a tumor suppressor with DNA repair function through homologous recombination in response to DNA double-stranded breaks. *BRCA1* deficiency results in genomic instability and individual’s susceptibility to breast and ovarian cancer [20–22]. Recently, Green and colleagues reported that low *BRCA1* expression was associated with high numbers of CD8^+^ TILs and poor survival in patients with breast cancer [23].

The process and presentation of peptide antigens in antigen-presenting cells include peptide cleavage, peptide transportation to membrane and its interaction with major histocompatibility complex (MHC) class I molecules. Recently, Goel and colleagues [24] reported that cyclin-dependent kinases CDK4/6 inhibitors triggered anti-tumor immunity by upregulating the expression of genes involved in the process and presentation of peptide antigens. Cyclin D1 (CCND1) is a regulatory cofactor of CDK4/6, and is overexpressed in breast cancer [25]. It has been shown that *CCND*1 and MHC-I molecules are negatively correlated in breast cancer [24], suggesting CCND1 may modulate the presentation of peptide antigens, thereby affecting T cell activation.

We recently reported that high T cell activation score was positively associated with better survival in breast cancer patients [26]. Given that impaired DNA repair such as deficiency in *BRCA1* results in genomic instability and susceptibility to genotoxic stress, and consequently leads to the increase of somatic mutations and neoantigens, we sought to answer the question whether the expression of *BRCA1* and *CCND1* modifies the effect of T cell activation on patients’ survival in breast cancer.

## Methods

### Study subjects

In this study, 1088 female patients with primary breast cancer, whose clinical data were retrieved from a TCGA dataset, were included. The average age at diagnosis was 58.3 (range: 26–90) years old. 75.7% (752 of 994) patients were Caucasians. Among the 1088 patients, 64.8% were post-menopause women, 21.1% pre-menopause, 3.7% peri-menopause, 3.1% indeterminate, and 7.3% unknown. Ductal carcinoma accounted for 71.8% (*n* = 780), followed by 18.6% lobular (*n* = 202), 4.9% mix (*n* = 53), and 4.7% other (*n* = 51). Majority of patients were diagnosed with breast cancer at early stage (181 (16.8%) stage I, and 616 (57.2%) stage II), and 26.0% (*n* = 280) at advanced stage (III or IV). Among the 1038 women with a known estrogen receptor (ER) status, 77.1% were ER-positive. Among the 719 patients with a known HER2 status, 22.4% were HER2-positive. Among the 1035 patients with a known progesterone receptor (PR) status, 67.1% were PR-positive. There were 520 women with molecular subtype information; 70.6% were luminal, 22.3% were basal-like, and 7.1% were HER2-enriched. The average follow-up in the 1086 patients with available follow-up information were 27.5 (range: 0–282.7) months. During the follow-up period, 154 patients died.

### BRCA1, CCND1, MHC-I, T cell activation- and exhaustion- related genes

We retrieved the upper quartile normalized RNA-seq by Expectation Maximization (RNA-seq V2 RSEM) data from a TCGA breast invasive carcinoma study available at TCGA provisional (https://www.cbioportal.org/) accessed as of July, 2018 [27, 28]. Expression data of *BRCA1*, *CCND1*, MHC-I, antigen-presenting-related genes, and 13 genes related to T cell activation status (8 genes associated to T cell activation: *NKG7*, *CCL4*, *CST7*, *PRF1*, *GZMA*, *GZMB*, *IFNG* and *CCL3,* and 5 genes associated to T cell exhaustion: *PD-1*, *TIGIT*, *LAG3*, *TIM3* and *CTLA4*) were available [9]. Experimental data generation and processing were previously described [29]. Gene expressions and clinicopathologic data were integrated, and the 1088 women patients with gene expression data were included in this study. No patients received neoadjuvant treatment.

### Statistical analyses

A weighted T cell activation score was calculated for each subject based on 13 genes relevant to T cell activation status as described previously [26]. The overall survival was the time from surgery until death. Spearman correlation was used to evaluate correlations.

To investigate whether the *BRCA1* and *CCND1* expression modifies the effect of T cell activation status on patients’ survival in breast cancer, we performed multivariate Cox proportional hazards models stratified by the *BRCA1* or *CCND1* level, where medians of the *BRCA1* and *CCND1* expression levels were used as cutoff values. Three groups, high (activation), intermediate, and low (exhaustion), were defined based on the T cell activation score as previously described [26]. Patient’s age at diagnosis, disease stage and histological type were included in the models to estimate adjusted hazards ratios (HRs) and their 95% confidence intervals (95% CIs). In the whole sample, we also assessed the interaction between T cell activation score and either *BRCA1* or *CCND1* levels by including their interactions in the Cox regression models. Proportional hazards assumption was examined. In all statistical analyses, a *p* value less than 0.05 was considered significant. Statistical analyses were performed using SAS version 9.4 (SAS Institute, Inc).

## Results

### Correlation between BRCA1 expression and T cell activation score

Table 1 shows the distribution of *BRCA1* expression and T cell activation score, and their spearman correlation. In 1088 tumor tissues, the average *BRCA1* expression level was 340.5 Fragments Per Kilobase of transcript per Million mapped reads (FPKM) (variation: 302.3 FPKM; range: 8.54–2267 FPKM). The median of the T cell activation score was 1.71 (range: − 40.7 - 251). The *BRCA1* expression was negatively correlated with the T cell activation score (correlation coefficient = − 0.13, 95% CI: -0.19 - -0.08, *p* < 0.0001).

**Table 1:** Distribution of the *BRCA*1 gene expression and T cell activation score, and their spearman correlation

Variable	N	Mean	SD^a^	Median	Range
*BRCA*1	1088	425.3	302.3	340.5	(8.5, 2267)
T cell activation score	1088	5.84	17.2	1.71	(− 40.7, 251)
Correlation coefficient (95% CI^b^)				−0.13	(−0.19, − 0.08)
*p*-value				< 0.0001	

^a^*SD* standard deviation

^b^*CI* confidence interval

### Correlation between CCND1 expression and, T-cell activation score, MHC-I and antigen-presenting related genes

The distributions of *CCND1*, MHC-I molecules (*HLA-A*, *B* and *C*) and the antigen-presenting related genes (*ERAP1* and *ERAP2* for peptide cleavage, *TAP1* and *TAP2* for peptide transportation, and *TAPBP* for transporter-MHC interactions) are shown in Table 2. *CCND1* was negatively correlated with T cell activation score (correlation coefficient = − 0.17, *p* < 0.0001), *HLA-A* (correlation coefficient = − 0.26, *p* < 0.0001), *HLA-B* (correlation coefficient = − 0.27, *p* < 0.0001), *HLA-C* (correlation coefficient = − 0.21, *p* < 0.0001), *ERAP2* (correlation coefficient = − 0.08, *p* = 0.01), TAP1 (correlation coefficient = − 0.26, *p* < 0.0001), *TAP2* (correlation coefficient = − 0.30, *p* < 0.0001) and TAPBP (correlation coefficient = − 0.16, *p* < 0.0001). No correlation was found between the expression levels of CCND1 and *ERAP1* (*p* = 0.109).

**Table 2:** Distribution of the *CCND1*, MHC-I and antigen-presenting related gene expression, and their spearman correlation

				*CCND1* (N = 1088)	
Variable	N	Median	Range	Correlation (95% CI)	*p* value
T cell activation score				−0.17 (− 0.22, − 0.11)	< 0.0001
*HLA-A*	1088	14,636	1290–186,441	− 0.26 (− 0.32, − 0.21)	< 0.0001
*HLA-B*	1088	22,385	2013–373,873	− 0.27 (− 0.33, − 0.22)	< 0.0001
*HLA-C*	1088	16,592	1455–224,187	− 0.21 (− 0.26, − 0.15)	< 0.0001
*ERAP1*	1088	1416	94–7157	0.05 (− 0.01, 0.11)	0.109
*ERAP2*	1088	643	4.5–6506	−0.08 (− 0.14, − 0.02)	0.0098
*TAP1*	1088	2259	205–41,853	− 0.26 (− 0.31, − 0.20)	< 0.0001
*TAP2*	1088	1148	117–15,665	−0.30 (− 0.35, − 0.24)	< 0.0001
*TAPBP*	1088	5193	1307–24,371	−0.16 (− 0.21, − 0.10)	< 0.0001
*CCND1*	1088	7925	129–245,328		

### Interaction between T cell activation status and either BRCA1 or CCND1 expression level in patient survival

In all the subgroups considered, the assumption of proportional hazards was verified based on 1000 simulations when testing the proportional hazards assumption (*p* = 0.073 in the low *BRCA1* group, *p* = 0.381 in the high *BRCA1* group, *p* = 0.688 in the low *CCND*1 group, and *p* = 0.758 in the high *CCND*1 group).

**Fig. 1:**
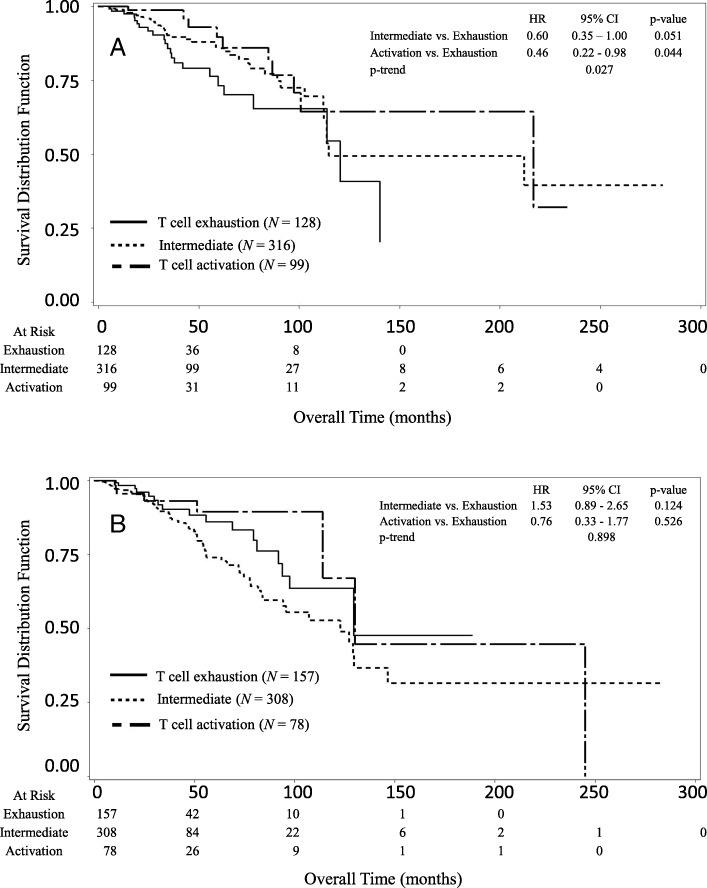
Kaplan-Meier survival curves of breast cancer patients stratified by the T cell activation status (score). **a** In the subgroup with a low *BRCA1* expression level, patients in the Activation group had better overall survival compared to those in the Exhaustion group (p-trend = 0.027). **b** In the subgroup with a high *BRCA*1 expression level, there were no significant difference in overall survival between patients in the Activation and Exhaustion groups (p-trend =0.898)

We investigated the relationship between survival and the T cell activation status (Activation, Intermediate, and Exhaustion) stratified by either the *BRCA1* or *CCND*1 expression levels. In the low *BRCA1* expression group, patients with a high T cell activation score (Activation) showed better overall survival compared to those with a low T cell activation score (Exhaustion) (Fig. 1a). The median of overall survival was 120.5 months (95% CI: 77.1 - ∞) in the Exhaustion group, 114.7 months (95% CI: 112.3 - ∞) in the Intermediate group, and 216.6 months (95% CI: 97.4 -∞) in the Activation group, respectively. Patients in the Activation group lived on average 96.1 months longer than those in the Exhaustion group. The Activation group showed significantly decreased risk of death (p-trend = 0.027) as compared to the Exhaustion group. The HRs of death were 0.60 (95% CI: 0.35–1.00) for Intermediate vs Exhaustion, and 0.46 (95% CI: 0.22–0.98) for Activation vs Exhaustion. In contrast, among the patients with a high *BRCA1* expression level, there was no significant difference in the overall survival between the Activation and Exhaustion groups (Fig. 1b). The median of overall survival was 129.5 months (95% CI: 93.8 - ∞) for the Exhaustion group, 122.7 months (95% CI: 83.8–146.4) for the Intermediate group, and 130.1 months (95% CI: 114.1–244.9) for the Activation group, respectively. Patients in the Activation group lived on average 0.6 months longer than those in the Exhaustion group. The HRs of death were 1.55 (95% CI: 0.89–2.65) for Intermediate vs Exhaustion, and 0.76 (95% CI: 0.33–1.77) for Activation vs Exhaustion, respectively. No significant trend in the risk of death was found for the T cell activation status in the patients with a high *BRCA1* expression level (p-trend =0.898). In the whole sample, the interaction test suggested a significant interaction between *BRCA1* expression level and T cell activation status (*p* = 0.043).

**Fig. 2:**
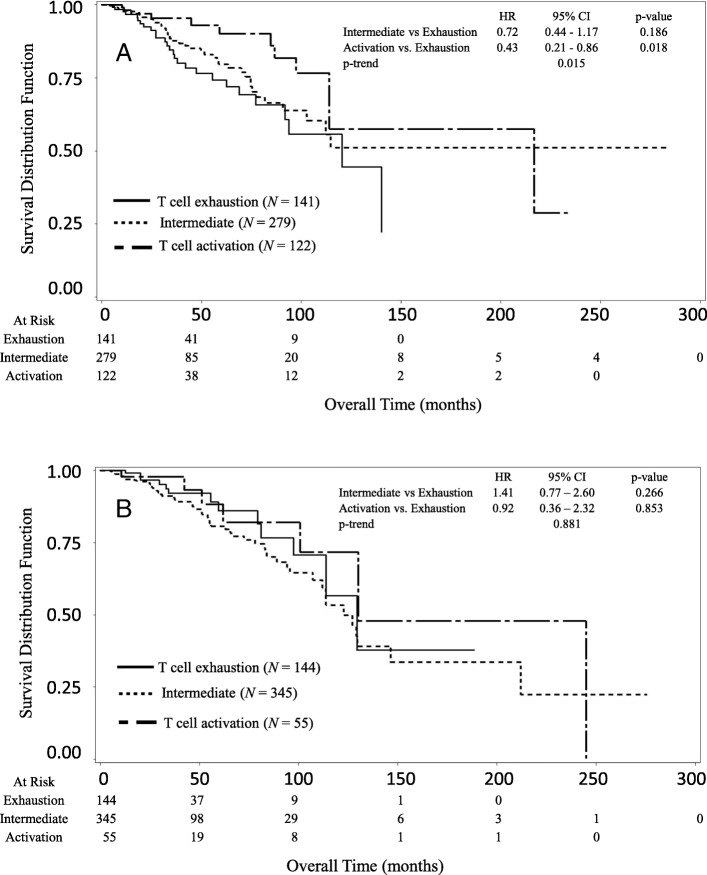
Kaplan-Meier survival curves of breast cancer patients stratified by the T cell activation status (score). **a** In the subgroup with a low *CCND1* expression level, patients in the Activation group had better overall survival compared to those in the Exhaustion group (p-trend = 0.015). **b** In the subgroup with a high *CCND1* expression level, there were no significant difference in overall survival between patients in the Activation and Exhaustion groups (p-trend = 0.881)

Figure 2 shows the Kaplan-Meier survival in three groups based on patients’ T cell activation status stratified by the *CCND*1 expression levels (low and high). In the low *CCND1* expression group, improved overall survival was observed for patients in the Activation vs. the Exhaustion group (Fig. 2a). The median of overall survival was 120.5 months (95% CI: 91.9 - ∞) for the Exhaustion group, the median survival did not reach (95% CI: 102.7 - ∞) for the Intermediate group, and 216.6 months (95% CI: 114.1 – ∞) for the Activation group, respectively. Patients in the Activation group lived on average 96.1 months longer than those in the Exhaustion group. The Activation group showed significantly decreased risk of death (p-trend = 0.015) as compared to the Exhaustion group. The HRs of death were 0.72 (95% CI: 0.44–1.17) for Intermediate vs Exhaustion, and 0.43 (95% CI: 0.21–0.86) for Activation vs Exhaustion. In contrast, in the patients with a high *CCND1* expression level, there was no significant difference in the overall survival between the Activation and Exhaustion groups (Fig. 2b). The median of overall survival was 129.5 months (95% CI: 97.4 - ∞) for the Exhaustion group, 122.7 months (95% CI: 112.0–146.4) for the Intermediate group, and 130.1 months (95% CI: 100.6–244.9) for the Activation group, respectively. The Activation group lived approximately 0.6 months longer than the Exhaustion group. The HRs of death were 1.41 (95% CI: 0.77–2.60) for Intermediate vs Exhaustion, and 0.92 (95% CI: 0.36–2.32) for Activation vs Exhaustion, respectively. No significant trend in the risk of death was found for the T cell activation status in the patients with a high *CCND1* expression level (p-trend =0.881). In the whole sample, there is no significant interaction between *CCND1* expression level and T cell activation status (*p* = 0.143).

We then performed multivariate Cox proportional hazards models to adjust for potential confounding variables including patient’s age at surgery, disease stage, tumor grade and histological types. Results are shown in Tables 3 and 4. Similarly, the association remained significant between the T cell activation status and death risk in the patients with a low *BRCA1* expression level (Table 3). The adjusted HRs were 0.47 (95% CI: 0.28–0.80) for Intermediate vs Exhaustion, and 0.43 (95% CI: 0.20–0.93) for Activation vs Exhaustion, respectively. T cell activation score was negatively associated with the death risk (p-trend = 0.011). In contrast, among the patients with a high *BRCA1* expression level, there was no significant association or linear relationship (p-trend = 0.640) between the T cell activation status and death risk. The adjusted HRs were 1.47 (95% CI: 0.85–2.55) for Intermediate vs Exhaustion, and 0.53 (95% CI: 0.20–1.43) for Activation vs Exhaustion, respectively. Again, the interaction test in the whole sample showed a significant interaction between *BRCA1* level and T cell activation status (*p* = 0.009) after adjustment for the covariates. Similarly, among the patients with a low *CCND*1 expression level, a high T cell activation score decreased mortality risk (Table 4). The adjusted HRs were 0.67 (95% CI: 0.41–1.10) for Intermediate vs Exhaustion, and 0.39 (95% CI: 0.19–0.81) for Activation vs Exhaustion, respectively. T cell activation score was negatively associated with the death risk (p-trend = 0.008). In contrast, among the patients with a high *CCND1* expression level, there was no significant association or linear relationship (p-trend = 0.882) between the T cell activation status and death risk. The adjusted HRs were 1.34 (95% CI: 0.73–2.47) for Intermediate vs Exhaustion, and 0.91 (95% CI: 0.33–2.52) for Activation vs Exhaustion, respectively. Similarly, the interaction test in the whole sample showed no significant interaction between *CCND1* level and T cell activation status (*p* = 0.135) after adjustment for the covariates.

**Table 3:** Association of the T cell activation status and mortality of breast cancer stratified by the *BRCA1* expression level

Stratification		Death		
Variable	Variable	HR^a^	95% CI^b^	*p*-value
Low *BRCA1*	T cell activation			
	Exhaustion	1.00		
	Intermediate	0.47	0.28–0.80	0.006
	Activation	0.43	0.20–0.93	0.031
	p-trend		0.011	
	Age (per 5 years)	1.15	1.05–1.27	0.004
	Disease Stage	2.78	1.89–4.10	< 0.0001
	Histological type			
	Ductal	1.00		
	Lobular	0.54	0.29–1.00	0.048
	Mix	0.81	0.29–2.28	0.683
	Other	2.36	0.92–6.03	0.074
High *BRCA1*	T cell activation			
	Exhaustion	1.00		
	Intermediate	1.47	0.85–2.55	0.174
	Activation	0.53	0.20–1.43	0.211
	p-trend		0.640	
	Age (per 5 years)	1.23	1.13–1.35	< 0.0001
	Disease Stage	2.07	1.45–2.96	< 0.0001
	Histological type			
	Ductal	1.00		
	Lobular	0.77	0.39–1.52	0.448
	Mix	0.68	0.25–1.87	0.450
	Other	2.79	1.09–7.13	0.032
*P*-value for the interaction between *BRCA1* level and T cell activation			0.009	

^a^HR: adjusted hazard ratio, which was obtained from a multivariate Cox proportional hazards regression model

^b^*CI* confidence interval

**Table 4:** Association of the T cell activation status and mortality of breast cancer stratified by the *CCND1* expression level

Stratification		Death		
Variable	Variable	HR^a^	95% CI^b^	*p*-value
Low *CCND1*	T cell activation			
	Exhaustion	1.00		
	Intermediate	0.67	0.41–1.10	0.113
	Activation	0.39	0.19–0.81	0.011
	p-trend		0.008	
	Age (per 5 years)	1.14	1.04–1.25	0.008
	Disease Stage	2.77	1.89–4.07	<.0001
	Histological type			
	Ductal	1.00		
	Lobular	0.61	0.34–1.12	0.109
	Mix	1.44	0.57–3.62	0.441
	Other	3.01	1.32–6.83	0.009
High *CCND1*	T cell activation			
	Exhaustion	1.00		
	Intermediate	1.34	0.73–2.47	0.352
	Activation	0.91	0.33–2.52	0.854
	p-trend		0.822	
	Age (per 5 years)	1.26	1.15–1.38	<.0001
	Disease Stage	2.09	1.47–2.98	<.0001
	Histological type			
	Ductal	1.00		
	Lobular	0.69	0.35–1.36	0.281
	Mix	0.38	0.12–1.23	0.106
	Other	1.44	0.49–4.24	0.509
*P* value for the interaction between *CCND1* level and T cell activation			0.135	

^a^HR: adjusted hazard ratio, which was obtained from a multivariate Cox proportional hazards regression model

^b^*CI* confidence interval

## Discussion

In this study, we investigated the relationships between the *BRCA1* expression and the T cell activation score, between *CCND1* and MHC-I molecules and antigen-presenting related genes, as well as the interaction between either the *BRCA1* or *CCND1* expression and the T cell activation status in breast cancer patient survival. Based on data in a cohort of 1088 patients with primary breast cancer, our analysis results suggested that there was a negative correlation between the *BRCA1* expression and the T cell activation score. In the subgroup with a low *BRCA1* expression level, patients with a high T cell activation score lived 96.1 months longer than those with a low T cell activation score, whereas in the subgroup with a high *BRCA1* expression level, patients with a high T cell activation score survived 0.6 months longer than those with a low T cell activation score. *CCND*1 was negatively correlated with T cell activation score, the MHC-I molecules (*HLA-A*, *HLA-B*, *HLA-C*), peptide transporter genes *TAP1* and *TAP2* expression, TAP binding protein gene *TAPBP* and peptide cleavage gene *ERAP2* expression. These findings agree with the report by Goel and colleagues [24], suggesting that MHC-I and antigen presenting-associated genes are high in the low *CCND1* expression group, and more antigens may be presented. Furthermore, in the subgroup with a low *CCND*1 expression level, patients in the Activation group lived 96.1 months longer than those in the Exhaustion group. However, in the subgroup with a high *CCND*1 expression level, patients in the Activation group survived approximately 0.6 months longer than the Exhaustion group.

Genomic instability is a hallmark of human cancer and plays an important role in cancer initiation and progression. This instability leads to the susceptibility of genomic DNA to genotoxic stress, which in turn results in DNA damage, including single-base DNA sequence changes, and structural (insertion/deletion) and copy number abnormalities. If DNA damage occurs, cell cycle will arrest and subsequently triggers DNA damage repair systems. With a high fidelity repair for DNA damage, genome integrity can be preserved and thereby prevents the potential of cancer initiation. However, in case of deficiency in DNA damage repair pathways, alterations of DNA sequences can occur which drives aberrant expression of oncogenes and tumor suppressor genes, and consequently results in tumors. As a key player in the homologous recombination-based DNA repair pathway, *BRCA1* acts as a tumor suppressor via maintaining genome integrity. Both DNA mutations and epigenetic silence can lead to the loss of function of *BRCA1* which are associated with human cancer including breast [30–32]. Indeed, epigenetic perturbation is a risk factor in patient survival of diverse cancer types [33, 34]. Interestingly, both *BRCA1* promoter hypermethylated tumors and *BRCA*1 mutated tumors share similar characteristics such as low pRb expression and being associated with basal/triple-negative subtype of breast cancer [35]. Birkbak et al. [36] reported that a higher number of DNA mutations were observed in *BRCA1*-mutated tumors in comparison to *BRCA1*-wild type tumors. Alexandrov et al. [37] demonstrated that a large number of insertion and deletion with overlapping microhomology at break point junctions were strongly associated with *BRCA1* mutations in breast cancer. Nolan and colleagues showed that in *BRCA1*-mutated triple-negative breast cancers (TNBCs), both mutational loads and the numbers of TIL significantly increased with the accompanying elevation of PD-1 and CTLA4 expression compared to those in the *BRCA1*-wild type one [38]. They also found that the genotoxic cisplatin treatment augmented anti-PD-1/anti-CTLA4 immunotherapy in *Brca1*-deficient mice [38]. The elevated DNA mutations in *BRCA1*-deficient tumors is due to the low fidelity PolƟ/PARP1-based alternative non-homologous end-jointing pathways for double-stranded DNA damage repair [39, 40]. It has been reported that high neoantigene loads were associated with *BRCA1* mutation and microsatellite instability [41, 42]. These neoantigenes act as foreign peptides to activate host immune systems. Thus, the finding in this study that the *BRCA1* expression level was inversely correlated with the T cell activation score provides extensive clinical evidence, supporting the proof of concept that BRCA1 deficiency (either mutation- or hypermethylation-induced) leads to increased potential neoantigens, which consequently activate T cells. It will be interesting to further explore how a *BRCA1* mutation, given that the inherit *BRCA1* mutations significantly increase the lifetime risk of developing breast and/or ovarian cancer, affect the effect of T cell activation status on patient survival in breast cancer in future studies when the information is available.

After the processing of somatic mutation-derived neoantigens by antigen presenting cells (APCs), the complex of MHC class I molecules and neoantigen-peptides then primes and educates effector CD8^+^ T cells, then activates the effector cells. Neoantigens on tumor cells determine which activated CD8^+^ T cells are recognized and eliminate tumor cells. Low *BRCA1* results in hypermutation in tumor cells and generates more somatic alterations in DNA and protein sequences with antigen potential. The enhancement of antigen presentation, for example, using Calreticulin (CRT), increases immune response [43, 44]. The upregulation of antigen processing and presentation-related genes is enriched, and serves as an underlying molecular mechanism of the anti-tumor immunity triggered by the CDK4/6 inhibitors in the animal models [24]. In line with this proof of concept, we found that in patients with a low expression level of either *BRCA1* or *CCND1*, the T cell activation score is negatively associated with the risk of mortality in breast cancer. Over 50% reduction in the risk of mortality (HR = 0.47 in patients with a low *BRCA1* expression level) was observed in patients with an intermediate T cell activation score, and in patients with a high T cell activation score (HR = 0.43 in patients with a low *BRCA1* expression level; HR = 0.42 in patients with a low *CCND1* expression level), compared to those with a low T cell activation score. Patients in the Activation group survived over 8 years longer than those in the Exhaustion group if they had a low level of *BRCA1* expression. In contrast, in patients with a high level of *BRCA1* expression, there was no significant association between the T cell activation score and the risk of mortality. The median survival were similar for three subgroups, Exhaustion, Intermediate and Activation. This finding suggests that the increase of neoepitopes (neoantigens) and the enhancement of antigen presentation can be utilized in immunotherapies in combination with immune checkpoint blockade, it could improve patient survival. The results from other studies and ours show that PLGA-nanoparticle-mediated delivery of tumor antigenic peptides effectively induce cytotoxic T cell responses and destroy tumor cells [45–48]. Virus-based tumor antigen delivery has been also extensively investigated. Osada and colleagues [49] recently demonstrated that recombinant adenoviral vectors encoding human HER3 substantially induced the TILs in tumors, elicited HER3-specific T cells and influenced the host response to immune checkpoint blockers.

Using the Genomics-driven immunoproteomics (GDI) approach that combines deep genomic sequencing and personalized immune assessment platform, 149 tumor antigens were discovered from breast cancer patients [50]. These putative neoantigens were derived from single nucleotides mutations, insertion and deletion. The high-throughput next-generation genome sequence in combination with personalized peptide array may be able to expedite tumor-associated antigenic peptide identification, and help design novel effective cancer vaccines by delivering tumor-specific neoepitopes to improve the efficacy of immune checkpoint-based immunotherapies. Recently, two clinical trials were carried out to evaluate the efficacy of tumor-specific neoantigens in patients with melanoma [51, 52]. The neoantigens were synthesized long peptides, which were predicted tumor-derived epitopes with a high affinity to bind MHC class I molecule based on a somatic mutation-related novel protein sequences using an algorithm. They found that the neoantigens activated effector T cells and substantial immune responses were observed in patients. Over 50% of patients (4 of 6 and 8 of 13, respectively) showed tumor free throughout the follow-up period (32 months and 12–23 months, respectively) after the vaccination. Tumor recurrence occurred in the remaining patients in both studies, however, who had well responded to the PD-1 inhibitor.

Studies have shown that TILs are one of the favorable prognostic markers in triple-negative or HER2^+^ breast cancer patients, but not in those with ER^+^/HER2^−^ [53–57]. Tomioka and colleagues reported that unfavorable prognosis was observed in triple-negative breast cancer with low TILs and high PD-L1 [58]. Understanding the factor(s) stimulating TILs may help to design novel strategies in switching ‘cold-’ to ‘hot-’ tumors. Recently, animal models showed that the RNA-editing induced RNA structure switching from single-strand RNA (ssRNA) to dsRNA could increase tumor inflammation and thereby overcome PD-1 blockade resistance [59]. The number of TILs significantly increased in BRCA1-mutated breast cancer tumors than the wild-type ones [38], suggesting that loss of *BRCA1* function might elicit tumor inflammation. However, the underlying mechanism(s) is yet to be determined. Given that the abundance and compositions of TILs are important in cancer immunotherapy, it warrants to further investigate how the TIL abundance and composition affect T cell activation and what factor(s) influences its abundance and composition.

## Conclusions

This is the first study to demonstrate the association of the *BRCA1* expression level and the T cell activation score, and their interaction in patient survival in breast cancer. The T cell activation score was negatively associated with the *BRCA1* expression and the *CCND*1 expression and the expressions of MHC-I molecules and antigen presenting related genes. In the subgroup of patients with low but not high levels of either *BRCA1* or *CCND1* expression, high T cell activation score significantly reduced the risk of mortality. These findings suggest that immune checkpoint inhibitors will benefit patients by reinvigorating effector T cells if they are either *BRCA1*-deficient or *CCND1*-low. Inhibition of *BRCA1* and *CCND1* genes may improve immune response by increasing neoantigens and their presentation. Tumor-specific neoantigen vaccine therapy and their efficient presentation may enhance patients’ response to immune checkpoint therapies. More studies with a larger sample size are warranted to further examine how the different molecular subtypes of breast cancer affect the interaction of *BRCA1* and *CCND1* with T cell activation in patient survival.
